# Ten years’ of Healthy Life Centers – research and directions for future work

**DOI:** 10.1177/14034948221081640

**Published:** 2022-03-18

**Authors:** Gro Beate Samdal, Eivind Meland

**Affiliations:** 1 VID Specialized University, Faculty of Health, Norway; 2 Department of Global Public Health and Primary Care, University of Bergen, Norway

## Background

The Norwegian strategy to prevent non-communicable diseases (NCDs) has placed strong emphasis on individual counselling for change in health behaviors. Healthy Life Centers (HLCs) are now established in more than half of our municipalities. These centers are similar to what other European countries have called Exercise Referral Schemes (ERSs). The research evidence behind the development of this public health measure was both insufficient and conflicting, but we now have more than 10 years’ experience and research from Norwegian HLCs. What does this research tell us, and what consequences should this research entail?

Healthy Life Centers (HLCs) have now been established in more than 60% of Norwegian municipalities and, during 2016, 27,000 participants attended one or more of the interventions offered at these primary health care facilities [1]. According to the Norwegian Directorate of Health, the beneficial effects of these interventions could be sustained for at least 1 year after HLC attendance. This success story, together with economic incentives from the government over several years, speeded up the implementation of HLCs across Norway [2]. In 2013, the Ministry of Health launched its Non-Communicable Disease (NCD) strategy, which also had a strong emphasis on individual counselling for behavior change [3].

The evidence for the effects of HLCs, however, was at that time both insufficient and conflicting [4,5]. A Norwegian review also concluded that any long-term effect was dubious [6]. Against this background, we set out to perform a meta-analysis of the short- and long-term effects of lifestyle interventions among overweight and obese adults who attended exercise and dietary interventions [7]. Contrary to some former reviews [4], we confirmed that the interventions were effective, in both the short-term (⩽6 months), and also the long-term (⩾12 months). We complemented the meta-analyses with meta-regression analyses to identify intervention characteristics associated with success. We will come back to these findings under the heading “Predictors explaining lifestyle improvements.”

The government’s recommendations for HLCs aim to promote physical and mental health, prevent or limit the development of disease, and contribute to mitigating health disparities [8]. Accordingly, a dual objective with emphasis both on physical health and mental wellbeing is important.

## Mitigating social health disparities

The Norwegian NCD strategy and the government’s recommendations for HLCs aimed to mitigate social health differences and recruit participants from socioeconomically disadvantaged groups [2,3]. Several studies show that the second objective was achieved. Included among HLC participants are those with low levels of education and income, people on long-term sick leave, and people with social and emotional challenges [9–11]. Conspicuously high levels of psychological distress were revealed in one study [12].

Qualitative studies among participants at HLCs show that the ability to improve health behavior may be hampered by adverse life experiences but, at the same time, improvements in health behavior may boost pride and mastery [13,14]. Participants who had accomplished 1 year follow-up reported that autonomous motivation and a supportive environment that promoted emotional coping and self-regulation of behavior were important [15]. These findings corresponded well with a qualitative interview study among health care professionals at the HLCs [16].

Drop-out rates may indicate social differentiation, although the findings from HLC studies are conflicting in this regard. In one study, a low level of education did not explain drop-out, which was more likely among participants with chronic somatic disease and mental or musculoskeletal challenges [17]. Another study found no socioeconomic predictors for dropping out, although this study had inadequate statistical power [18]. In a third study, non-attendance among invited participants at risk of NCDs had a strong socioeconomic link, but only among women (low educational level) [19].

In a randomized controlled trial (RCT) performed at HLCs in western and southern Norway, changes in rates of moderate and vigorous physical activity (MVPA) were slightly more favorable among more highly educated participants during 6 months’ follow-up, compared with participants with medium-to-low levels of education [17]. The intervention had no overall effect when compared with the control group. Interaction analyses, however, revealed an increase in physical activity (PA) for those least active in the intervention group compared with the least active controls. In the same study, educational attainment did not differentiate change in weight or body attitude. The intervention and baseline weights interacted, however, disfavoring those with heavy bodies [20].

Participants’ level of education (high versus low) could not explain PA improvements in a 3-month observation study encompassing greater numbers of participants from multiple HLCs [21]. The same study revealed that not only did participants with mental or somatic diseases and with excess weight improve their PA level, the same was true of their peers. In the RCT cited above, participants’ level of education could not explain a change in healthy eating after 6 months. However, gross family income had a paradoxical impact on unhealthy eating, favoring those with lower income [22].

Based on these results, we may conclude that, although socioeconomic level is related to health and health behaviors at a population level, the HLC interventions do not seem to reinforce social inequalities in health. On the other hand, we have no indications from the HLC research that health disparities are mitigated. The interventions are part of a preventive strategy for individuals at risk for future NCDs. As such, we cannot expect a great influence on health at the population level, even though individuals at high risk may benefit. Population strategies with systemic efforts in communities, in workplaces, in schools, and leisure time activities are called for to improve population health and mitigate health disparities [23]. Studies comparing systemic interventions with individually tailored promotive efforts are needed.

## Does it work, for whom, and for how long?

The answers to the questions “Does it work, for whom, and for how long?” depend on what outcomes we examine. The Norwegian Directorate of Health’s guidelines for HLCs have increasingly emphasized a holistic approach in this health care service [8], but PA has been the main focus. However, the evidence for this outcome is limited by methodological shortcomings. The RCT cited above had limited external validity due to low uptake among those invited, favoring already active individuals [17]. The observation study from multiple HLCs had limited internal validity because the study lacked a control group, and we cannot discern intervention effects from secular trends and other unspecific factors [10].

In the RCT, the intervention and control groups experienced similar and nonsignificant differences in MVPA change during 6 months. Interaction analyses revealed that less physically active participants in the intervention group improved their MVPA, when compared with similar participants in the control group [17]. The observation study discerned an improvement in PA measured with accelerometers after 3 months, but the improvement was lost after 15 months [10]. Preliminary results from observational data during 6 and 24 months in the RCT study revealed similar findings (publication in progress).

In the longitudinal observation study, health-related quality of life (QoL), including limitations due to emotional problems and mental health in general, improved 3 months after baseline, and the change was maintained after 15 months [10]. The researchers revealed that QoL improvements were related to participants’ PA changes [10]. This finding is in accordance with findings from the British exercise referral schemes, demonstrating effects on anxiety and depression for those referred for mental health problems, whereas improvements of PA were restricted to those suffering from cardiac disease [24]. In the RCT, healthy eating improved significantly only among participants in the intervention group who accepted a healthy eating intervention in addition to PA intervention [22].

The majority of participants at the HLCs wanted help with their body weight, and weight is measured at most HLCs [9]. The RCT could not reveal any weight difference between the control and intervention groups. However, interaction analyses showed that participants with leaner bodies in the intervention group experienced weight loss when compared with leaner participants in the control group [20]. A generally expected and unwanted side effect of body weight focus is stigmatization, body concern, and body attitude problems [25]. Although, we revealed no such side effects in the RCT [20], we cannot rule out that health promotive efforts with a focus on body weight can contribute to stigmatization and social marginalization.

## Predictors explaining lifestyle improvements

In the meta-analytic study, we identified behavior change techniques (BCTs) applied in diet and PA interventions for participants that resemble the participants at the HLCs. We sought to reveal how the techniques could explain variations in intervention effects between the studies. We revealed that self-regulation skills such as goal setting and self-monitoring of behavior explained both short-term (⩽6 months) and long-term intervention success (⩾12 months) [7]. Using the BCTs goal setting of outcome, feedback on outcome of behavior, implementing graded tasks, and adding objects to the environment, e.g., using a step counter, significantly predicted maintenance of long-term change.

In addition, interventions emphasizing autonomy support and person-centeredness in counseling also explained long-term intervention effects. These findings align well with the qualitative studies cited above [15,16]. Autonomy support and care for the individual are important, but just as important is a counselling approach that supports self-regulation skills. In the context of HLCs it seems relevant that counselling for psychological challenges should be emphasized for many participants [15].

In the 6-month RCT, we examined several motivational factors with an expected yield on health behavior change. We were unable to identify significant impact on MVPA change from any of these factors. The study on body mass and body attitude revealed that some factors that impacted weight loss also predicted body attitude deterioration. However, higher levels of self-rated health (SRH) and autonomous motivation impacted weight loss. SRH simultaneously predicted improvement in body attitude. A beneficial body attitude was also predicted by life satisfaction and self-efficacy for PA [20]. In a separate study, we revealed that healthy eating may be improved by an emphasis on developing positive self-concepts like better SRH, vitality, life satisfaction, and self-esteem [22].

These findings support a holistic approach in health behavior counselling, in line with the intentions put forward in the updated recommendations from Norwegian health authorities [8]. An emphasis on positive individual and relational health promotive factors are important. It is, however, still unclear if the effectiveness and cost-effectiveness of this healthcare measure is clearly documented.

HLC services are not mandated by law in Norwegian municipalities, and the services vary according to local contexts and available resources [26]. Effects vary according to study site [10], and lack of standardization leaves us with insecurity about which elements contribute to the effect [27]. We must, however, acknowledge that standardization of complex interventions has a trade-off in terms of local ownership and local competence. Therefore, multifaceted research methods with different outcomes, different intermediate and pathway variables, and a combination of qualitative and quantitative data are called for [28]. The Norwegian HLC studies satisfy many of these preconditions but, like the British ERSs, we still lack knowledge about what works for whom [24].

## Theory-informed and evidence-based practice

It is a complicated task to develop healthcare services that are theory-informed and evidence-based. Health behavior researchers admit that knowledge is insufficient on how lifestyle changes can be promoted at a population level [29,30]. Development in behavior sciences in recent years can help us better understand human behavior and promote effective interventions [29,31]. Three elements are called for: (1) theoretical knowledge and scientific evidence, (2) acknowledging the importance of contexts for behavior and health, and (3) translating research evidence to clinical practice [32].

The recommendations for HLCs emphasize that the services should be based on evidence, and this is also expected from local stakeholders in communities [8,33]. A patient-centered and salutogenic perspective is recommended, and counselling should build upon motivational interviewing (MI), supplemented with techniques from cognitive behavior therapy (CBT) [8]. However, the developers of MI—William Miller and Stephen Rollnick—recommend a multifaceted approach. MI is important in dealing with ambivalence and revealing the individual’s personal motivation for change [34]. The quality of the motivation, if it is regulated for autonomous reasons or regulated externally, is decisive for long-term maintenance of health behavior [35]. Self-regulation of behavior is important, but energy depleting in the long run. Internal motivation, the experience that the behavior is joyful and important for personal reasons, needs less energy and is self-perpetuating [36].

The BCT framework referred to above has gained international recognition [37]. The framework enables a common language for (1) development and quality assurance of interventions, (2) evaluating interventions, and, not least, (3) synthesizing research evidence about effective techniques in interventions. We lack knowledge about what BCTs Norwegian HLC providers use in their counselling, except that the recommendations urge using client-centered methods, such as MI and CBT. A fidelity study among British ERSs showed that BCTs with poor evidence were often applied, for instance information giving, while techniques with stronger evidence were seldom used, e.g., self-monitoring of behavior [38].

## Where to go?

We suggest some improvements for further development of HLC services. First, health authorities can provide a systematic framework and program theory for how the services are meant to function, based on behavior science [30,31]. A program theory describes why a goal in a certain context has effect and includes both formal scientific theory and local experience. Such a description can optimize counselling and identify elements with both short-term and long-term efficacy [31,39].

Second, we can learn from other countries. The United Kingdom’s National Institute for Health and Care Excellence (NICE) has both a guideline for the ERS services and a guidelines for individual counselling [40,41]. Scottish health authorities have developed a hierarchy for counselling competence. Their goal is to promote all health personnel with a basic competence for short interventions. Basic competence can, thereafter, be developed to advanced competence in more extensive and long-lasting counselling for behavior change [42]. Swedish guidelines build on a similar cumulative hierarchy [43].

Third, our health authorities can establish a national database to collect health and QoL data before and after participation at the HLCs, similar to what NICE has initiated in the National Referral Database [44]. Standardized data collection across all the HLCs may enable us to identify success factors, to evaluate the results, and to translate the research evidence to clinical practice.
